# Bortezomib-based Regimens Improve the Outcome of Patients with Primary or Secondary Plasma Cell Leukemia: A Retrospective Cohort Study

**DOI:** 10.4274/tjh.galenos.2019.2019.0254

**Published:** 2020-05-06

**Authors:** Huijuan Wang, Huixing Zhou, Zhiyao Zhang, Chuanying Geng, Wenming Chen

**Affiliations:** 1 Beijing, China; 2 Workers Stadium South Road, Chaoyang District, Beijing, China; 3 Chaoyang District, Hematology, Beijing, China Beijing

**Keywords:** Primary plasma cell leukemia, Secondary plasma cell leukemia, Bortezomib-based treatment, Overall survival

## Abstract

**Objective:**

The management experience for plasma cell leukemia (PCL) is still limited by PCL’s rare incidence and aggressive course. The goal of this study was to further identify the efficacy of bortezomib-containing regimens for PCL in Chinese patients.

**Materials and Methods:**

In this study, 56 consecutive PCL patients [14 primary PCL (pPCL) and 42 secondary PCL (sPCL) cases] were retrospectively enrolled and 42/56 patients received bortezomib-based regimens (BBRs), including 10/14 pPCL and 32/42 sPCL patients. The patients’ survival data, clinical information, and safety data were collected and analyzed.

**Results:**

In pPCL and sPCL patients, the overall response rate in the bortezomib group was 90.0% and 25.0%, respectively. The median progression-free survival from PCL diagnosis for pPCL and sPCL was 8.3 months vs. 2.9 months (p=0.043) and median overall survival (OS) from PCL diagnosis was 23.3 months vs. 4.0 months. The OS for patients receiving BBRs was significantly longer for both pPCL (8.3 vs. 1.2 months, p=0.002) and sPCL (4.3 vs. 1.1 months, p<0.001). In multivariate COX analysis, BBR treatment [p=0.008, hazard ratio (HR)=0.38, 95% confidence interval (CI)=0.19-0.77] and very good partial response or better (≥VGPR) (p=0.035, HR=0.19, 95% CI=0.04-0.74) were independent predictors of OS for sPCL patients. For pPCL patients, BBR predicted OS (p=0.029, HR=0.056, 95% CI=0.004-0.745) instead of ≥VGPR (p=0.272, HR=3.365, 95% CI=0.38-29.303).

**Conclusion:**

It was found that BBRs could significantly improve OS for both pPCL and sPCL patients.

## Abstract (tr)

**Amaç:**

Plazma hücreli lösemide (PHL) tedavi deneyimi PHL’nin nadir görülmesi ve agresif seyri nedeni ile halen sınırlıdır. Bu çalışmanın amacı Çin’deki PHL hastalarında bortezomib temelli tedavi rejimlerinin etkinliğini belirlemektir.

**Gereç ve Yöntemler:**

Bu çalışmaya geriye dönük olarak 56 PHL olgusu [14 birincil PHL (pPHL) ve 42 ikincil PHL (sPHL)] dahil edilmiştir ve pPHL 10/14 ve sPHL 32/42 olmak üzere 42/56 olgu bortezomib temelli tedavi (BTT) almıştır. Hastaların sağ-kalım verileri, klinik bilgileri ve güvenlik verileri toplandı ve analiz edildi.

**Bulgular:**

PPHL ve sPHL hastalarında bortezomib grubunda genel yanıt oranı sırasıyla %90 ve %25 idi. PHL tanısından itibaren ortanca hastalıksız sağ kalım pPHL ve sPHL için sırasıyla 8,3 ay ve 2,9 ay (p=0,043) ve genel ortanca sağkalım (GS) 23,3 ay ve 4 ay idi. BTT alan hastalar için GS hem pPHL (8,3 aya 1,2 ay, p=0,002) hem de sPHL (4,3 aya 1,1 ay, p<0,001) için anlamlı olarak daha uzun bulundu. Çok değişkenli COX analizinde BTT [p=0,008, kalp atım oranı (KAO)=0,38, %95 güven aralığı (CI)=0.19-0.77] ve daha iyi ya da çok iyi kısmi yanıt (≥ÇİKY) (p=0,035, KAO=0,19, %95 CI=0,04-0,74), sPHL hastaları için GS’ın bağımsız göstergesidir. PPHL hastalarında BTT için öngörülen GS (p=0.029, KAO=0,056, %95 CI=0,004-0,745) iken, ≥ÇİKY için (p=0,272, HR=3,365, %95 CI=0,38-29,303) idi.

**Sonuç:**

BTT’nin hem pPHL hem de sPHL hastalarında genel sağkalımı belirgin olarak iyileştirebileceği bulunmuştur.

## Introduction

Plasma cell leukemia (PCL) is the most aggressive disease among plasma cell malignancies with malignant plasma cells present in the peripheral blood, which accounts for 2%-4% of patients with multiple myeloma [1]. The diagnostic criteria of PCL are based on the presence of more than 20% plasma cells in the peripheral blood or an absolute plasma cell count of greater than 2x10^9^/L [2,3]. Primary PCL (pPCL) patients represent cases of de novo leukemia, accounting for 60% of PCL cases. Secondary PCL (sPCL) represents aggressive transformation of relapsed or refractory multiple myeloma (MM), occurring in 40% of PCL cases.

The survival of PCL patients remains considerably poor, especially for sPCL patients [1,4], and because of its low incidence and extreme aggressiveness, the therapeutic management of PCL is limited. Results from both retrospective [5,6] and prospective research [7,8] are insufficient and no explicit conclusion has been reached. The purpose of this study was to explore the survival of pPCL and sPCL patients being treated with bortezomib-based regimens (BBRs) in China.

## Materials and Methods

### Patients

We retrospectively and consecutively collected data of 56 PCL patients (including 14 with pPCL and 42 with sPCL) diagnosed and treated in Beijing Chao-Yang Hospital, Capital Medical University, between 2000 and 2017. Diagnosis of PCL was based on the criteria proposed by the International Myeloma Working Group (IMWG) [9].

### Methodology

We retrospectively collected clinical data of pPCL and sPCL patients during the aforementioned period of time. These clinical data included the date of pPCL or sPCL diagnosis, the date of last follow-up, progression-free survival (PFS), overall survival (OS), and information about the treatment. This study was conducted in accordance with the World Medical Association Declaration of Helsinki and approved by the Ethics Committee of Beijing Chao-Yang Hospital, Capital Medical University. The patients or relatives gave their written informed consent. Baseline data are shown in Table 1.

Response to treatment was evaluated according to the IMWG criteria [10]. BBRs were defined as triplet or quartet therapy containing bortezomib according to the IMWG consensus, administered subcutaneously at a dose of 1.0 to 1.3 mg/m^2^ once or twice a week.

### Statistical Analysis

One-way ANOVA, Pearson’s chi-square test, and the Mann-Whitney U test were used for the calculation of significant differences and correlations of clinical and laboratory features and response rates between groups. The Kaplan-Meier method was used to estimate survival curves. Cox regression univariate and multivariate analyses were used to measure possible independent predictive factors for survival. Values of p<0.05 were considered statistically significant. Statistical description and analysis were carried out with the software package IBM SPSS 24 (IBM Corp., Armonk, NY, USA).

## Results

### Patients

There were 56 PCL patients diagnosed and treated from 2000 to 2017 in Beijing Chao-Yang Hospital, Capital Medical University. Fourteen patients had pPCL (0.87% of all MM patients) and 42 patients had sPCL (2.61% of all MM patients). Five patients (35.7%) with pPCL and eight patients (19.0%) with sPCL were ≥65 years old. For sPCL patients, the median time from diagnosis of MM to progression to sPCL was 26.5 months (range=14.9 to 48.8 months). The baseline characteristics of the sPCL and pPCL groups are listed in Table 1. Platelet counts were significantly higher in pPCL (p=0.002). Lactate dehydrogenase (LDH) was significantly higher in sPCL (437.5 U/L vs. 166.3 U/L, p<0.05). Age and serum Ca and β_2_-microglobulin did not differ between pPCL and sPCL (p>0.05). Immunophenotyping data of the peripheral blood plasma cells were available for 37 of 59 patients and CD56 was negative in 15 of 37 (40.5%) patients. The frequency of CD20 and CD27 expression was significantly higher in pPCL patients than sPCL patients (21.4% vs. 7.1%, p=0.004; 35.7% vs. 7.1%, p<0.001).

Fluorescence in situ hybridization data were available for 24 patients; 16/24 patients (66.7%) presented with high-risk features including del17p present in 8 patients, t(4;14) present in 6 patients, and t(14;16) present in 5 patients (Table 1). In particular, 9 sPCL and 2 pPCL patients presented with 2 or 3 cytogenetic aberrations concurrently. The occurrence of del17p and t(14;16) was markedly higher in sPCL patients pPCL patients (19% vs. 7.1%, p=0.019; 0% vs. 11.9%, p<0.001), while the occurrence of t(4;14) was significantly higher in pPCL patients than sPCL patients (21.4% vs. 7.1%, p=0.007).

### Response to Treatment

Treatment regimens in patients with pPCL and sPCL are listed in Table 2. Conventional regimens are regimens without proteasome inhibitors and immunomodulatory drugs, including DECP (cisplatin, etoposide, cyclophosphamide, dexamethasone) and VMP (vincristine, melphalan, prednisone). The median treatment cycle number was 11 cycles in pPCL and 3 cycles in sPCL patients. Of the sPCL patients, 88.7% patients had novel drug-based induction therapy before progression to sPCL, and in total 42/56 (75.0%) patients (including 10 pPCL and 32 sPCL) received bortezomib-based induction for the treatment of PCL. Nine patients (2 pPCL and 7 sPCL) underwent autologous stem cell transplantation (ASCT). Overall response rate (ORR) was 71.4% in pPCL [complete response (CR)=21.4%, very good partial response (VGPR)=28.6%, partial response (PR)=21.4%, stable disease (SD)=21.4%, partial disease (PD)=7.1%) and 19% in sPCL (CR=4.8%, VGPR=2.4%, PR=11.9%, SD=45.2%, PD=35.7%).

ORR differed significantly between patients who received BBRs versus those who received conventional regimens (40.5% vs. 7.1%, p=0.044) (Table 3). Response rates significantly differed between patients who received BBRs and conventional regimens in both pPCL (90.0% vs. 25.0%) and sPCL (25.0% vs. 0%); pPCL patients who received a BBR had the highest response rate and the median time to progression for pPCL was 8.4 months (95% CI=2.4-10.9). The results demonstrated that bortezomib could improve the quality of response in both pPCL and sPCL patients.

### Survival Data

**Figure 1:**
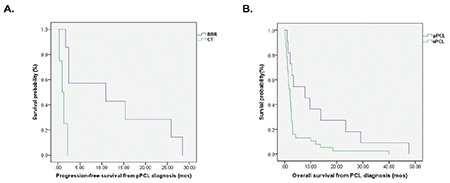
PFS and OS from PCL diagnosis in patients with primary PCL (pPCL) and secondary PCL (sPCL). PFS: Progression-free survival, OS: overall survival, PCL: plasma cell leukemia.

The median follow-up of the total 56 patients was 32.1 months (range=1.3-104.7 months). At the end of the follow-up time, 3 of 14 pPCL and 4 of 42 sPCL patients were alive. The median PFS from PCL diagnosis for pPCL and sPCL was 8.3 months vs. 2.9 months (p=0.043) (Figure 1A). The median OS from PCL diagnosis for pPCL and sPCL was 23.3 months (95% CI=4.1-21.6) vs. 4.0 months (95% CI=1.7-6.2) (p=0.012) (Figure 1B). sPCL patients were much more likely to experience disease progression during treatment.

**Figure 2:**
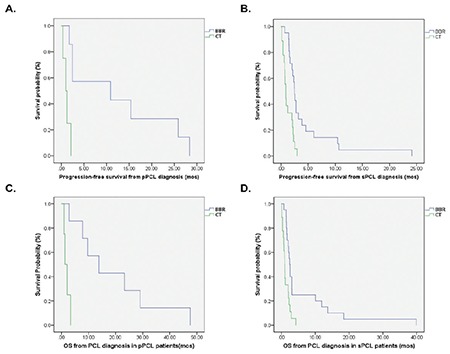
PFS and OS of patients treated with bortezomib-based regimens (BBRs) and conventional therapy (CT). A, B) pPCL PFS and sPCL PFS; C, D) pPCL OS and sPCL OS. PFS: Progression-free survival, OS: overall survival, PCL: plasma cell leukemia.

The median PFS in pPCL patients undergoing a BBR was significantly longer than that of those receiving conventional therapy (8.3 vs. 1.2 months, p=0.002), as was also the case for sPCL patients (4.3 vs. 1.1 months, p<0.001) (Figures 2A and 2B). Furthermore, BBR treatment also significantly improved OS in both pPCL patients (19.1 vs. 2.1 months, p=0.002) and sPCL patients (6.2 vs. 1.4 months, p=0.001) (Figures 2C and 2D). The median OS after relapse for pPCL and sPCL patients treated with BBR was 4.5 months and 1.6 months, respectively. There were 2 pPCL patients and 7 sPCL patients who received autologous hematopoietic stem cell transplantation (HSCT) therapy. The median OS of HSCT recipients was 29.1 months in pPCL patients and 17.5 months in sPCL patients. Furthermore, the OS for patients who achieved CR and VGPR was remarkably better than that of those who achieved PR or less in both pPCL (19.5 vs. 1.9 months, p=0.002) and sPCL (16.2 vs. 2.4 months, p=0.006).

Univariate Cox regression analysis showed that type of PCL, LDH, type of treatment (BBR vs. conventional treatment), and quality of response indicated significantly better OS from the PCL diagnosis (p<0.05). For pPCL, OS significantly benefitted from BBR and high-quality response (p=0.033, HR=6.877, 95% CI=1.173-40.322; p=0.040, HR=2.930, 95% CI=1.049-8.183, respectively). For sPCL patients, BBR treatment (p=0.001, HR=3.252, 95% CI=1.603-6.598) and high-quality response (≥VGPR, p=0.021, HR=1.937, 95% CI=1.1-3.4) also effectively contributed to OS. In multivariate COX analysis, BBR treatment (p=0.008, HR=0.38, 95% CI=0.19-0.77) and response ≥VGPR (p=0.035, HR=0.19, 95% CI=0.04-0.74) were independent predictors of OS for sPCL patients, while for pPCL patients, BBR predicted OS (p=0.029, HR=0.056, 95% CI=0.004-0.745) instead of ≥VGPR (p=0.272).

### Safety

sPCL patients constituted the majority of our population and most of them were exposed to bortezomib treatment. Therefore, there was a higher incidence of grade 3 and 4 adverse events for this mixed population. In bortezomib-treated patients, grade 3 or 4 myelosuppression was present in 48.2% of patients. Grade 3 or 4 neurotoxicity happened in 19.6% of patients. Gastrointestinal toxicity of grade 3 or 4 was present in 16.1% of patients. The incidence of grade 3-4 renal toxicity and hepatic toxicity was 8.9% and 12.5%, respectively. Neutropenic infection was present in 32.1% of patients, and seven patients died from acute respiratory failure caused by neutropenic infection in the bortezomib group.

## Discussion

PCL is an extremely rare and aggressive form of plasma cell malignancy [4], and the OS from diagnosis ranges from 7 to 14 months [11,12]. The survival of patients with pPCL is short. In seven series, the historical median survival without novel therapies ranged from 6.8 to 12.6 months in the era of conventional therapy [3,11,13,14]. Novel agents followed by stem cell transplant yielded prolonged survival of more than 3 years [15]. The best survival data, incorporating hematopoietic stem cell transplantation, demonstrated median survival of longer than 3 years [15]. However, in the era of novel agents, the proteasome inhibitor bortezomib has shown clinical efficacy in both pPCL and sPCL [16,17]. BBRs could improve both therapeutic response and survival of PCL patients, especially those with pPCL [5]. Furthermore, in the study by Katodritou et al. [18], bortezomib-based treatment showed clinical activity in pPCL patients with t(4;14) and CD27 expression. In another study by Katodritou et al. [6], with BBRs and a median follow-up of 51 months, the median OS of the patients with pPCL and sPCL treated with BBRs was 18 and 7 months, respectively. Autologous or allogenic HSCT has yielded encouraging outcomes and could prolong survival to more than 30 months [1,15,19]. However, only younger and highly eligible patients may benefit from stem cell transplantation and there are limited data from novel drug-based regimens in the treatment of PCL. The incidence of PCL is rare and the aggressively poor physical status of patients cannot tolerate the adverse effects of novel drugs. In recent years, however, several case series of PCL indicated that both pPCL and sPCL patients could benefit from bortezomib regimens [5,6,20,21,22,23].

Our current data collected from a single center are from 14 pPCL patients and 42 sPCL patients, representing the largest retrospective study with the longest follow-up time in China. To date, the largest series of pPCL treated with BBR was reported by Katodritou et al. [23], which included 50 pPCL patients, and that of Mina et al. [24], which enrolled 38 pPCL patients. The study of Jurczyszyn et al. [25] summarized the results of 101 sPCL patients. We have reported an ORR of 71.5% in pPCL patients receiving BBRs, which is similar to the result of 70% reported by Katodritou et al. [23]. However, our ORR is much higher than that of the previous study without novel agents. Meanwhile, Katodritou et al. [23] reported 100% ORR for pPCL patients with bortezomib-therapy and ASCT. As only 1 of our pPCL patients received allogenic HSCT treatment, our study cannot evaluate the role of bortezomib-therapy + allo-HSCT for pPCL patients, which is one of the deficiencies of this study.

For sPCL patients, bortezomib treatment could also contribute to higher ORR and prolong survival significantly. Our data are in accordance with the aforementioned studies, with a slightly lower overall response of 70.0% for ≥VGPR in pPCL patients. In sPCL patients treated with BBRs the ORR was 25%, which corresponds with the 36.4% ORR of Katodritou et al. [6] but is lower than the ORR of 60% reported by Jurczyszyn et al. [25].

With respect to survival, at the time of data collection, 3/14 (21.4%) pPCL patients and 4/42 (9.5%) sPCL patients receiving BBRs were still alive. Most sPCL patients die after the disease progresses. The median OS of PCL patients diagnosed with pPCL and sPCL was 23.3 months vs. 4.0 months, whereas the median OS of PCL patients diagnosed with pPCL and sPCL who received BBRs was 19.1 months vs. 6.8 months, respectively. Multivariate Cox regression analysis also proved BBRs to be positive predictors for both pPCL and sPCL patients, which highlights the impact of bortezomib treatment of PCL patents. Our conclusion is in accordance with previous studies. More remarkably, our data demonstrate that BBRs contributed to much longer OS for both pPCL and sPCL patients. However, because of the small number of pPCL patients, the survival data of our pPCL patients should be further validated by data from larger samples. In the study by D’Arena et al. [5], 2-year median follow-up reached 55% while median follow-up was not reached. In the multicenter retrospective study of Pagano et al. [14], the median OS for 73 pPCL patients was 12.6 months and HSCT patients had a longer OS (median=38.1 months). In our study, the median OS of HSCT-treated PCL patients was 29.1 months in pPCL patients (2/14 patients) and 27.53 months in sPCL patients (7/42 patients). Though the small number of patients limits the reliability, the results still highlight the benefits of HSCT.

The study of Lebovic et al. [21] reported the data of 25 PCL patients (13 with pPCL) treated with bortezomib-based agents and 19 patients received HSCT. The median OS of pPCL patients treated with a bortezomib-based agent was 28.4 months and the 18 patients treated with bortezomib regimens had the opportunity for optimum treatments, which could explain the better survival of those patients. In the study by Katodritou et al. [6], only six of the pPCL patients had undergone autologous HSCT and HSCT was not a significant predictor for OS in the univariate analysis. On the other hand, 45% of patients were still alive at 2 years, and after 4 years and 3 months of median follow-up 28% of all pPCL patients were still alive. The administration of “triplet” bortezomib-based treatment in 15/18 pPCL patients could probably explain the high ORR and the longer survival in their study.

In our study, according to multivariate COX analysis, treatment with BBRs and high-quality response (≥VGPR) positively predicted OS after PCL diagnosis. Likewise, in the studies of Katodritou et al. [6], Jurczyszyn et al. [25], and Mina et al. [24], it was reported that high-quality response was an important positive indicator of OS in pPCL patients. To some extent, BBRs and other novel agents may overcome the negative impact of highly aggressive PCL. Nevertheless, further verification is needed.

Bias on account of financial situation and comorbidities of patients also exists in this study, which is an inevitable factor in real-world clinical work. Our clinical features between the 2 groups were mostly matched. Because of the small sample of pPCL patients, the results will be further verified in a future study.

Safety is one of the important factors affecting the efficacy of bortezomib, especially in elderly myeloma patients. Our results showed that the adverse effects were acceptable even in sPCL patients who received BBRs for induction therapy, similar to the study of Katodritou et al. [6], in which grades 3/4 neurological, hematological, and infectious adverse events happened in 7%, 41.4%, and 31% of cases, respectively. In the study of D’Arena et al. [5], grades 3 and 4 hematological, neurological, and infectious events occurred in 20%, 21%, and 16%. As our study included more sPCL patients and older patients, our incidences of infection and neurological adverse events were relatively higher.

## Conclusion

Our data from a relatively high number of PCL patients have shown that treatment with BBRs is highly effective and safe in cases of PCL. BBRs and patients’ high-quality responses could be independent predictors for OS in PCL patients. BBRs are among the best therapeutic options for PCL patients, which could contribute to both therapeutic response and further overall survival. However, the defects of this study lie in the lack of data from ASCT PCL patients, which leads to weaker survival data than in other works. The conclusion is still required to be validated in studies with further large numbers of PCL patients. With novel drugs arising, new management approaches for both primary and secondary PCL will appear for deeper response and longer survival.

## Figures and Tables

**Table 1:**
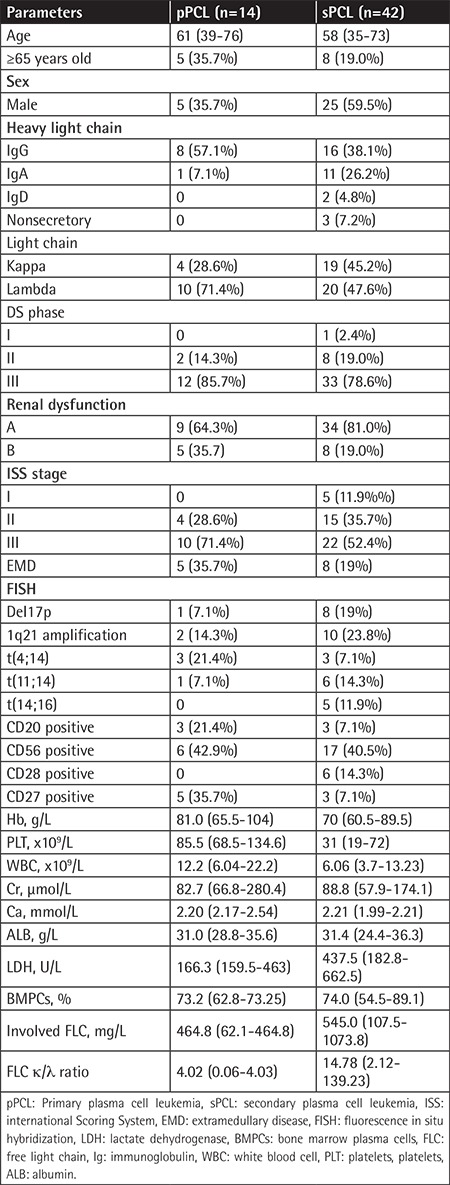
Patients’ characteristics of pPCL and sPCL.

**Table 2:**
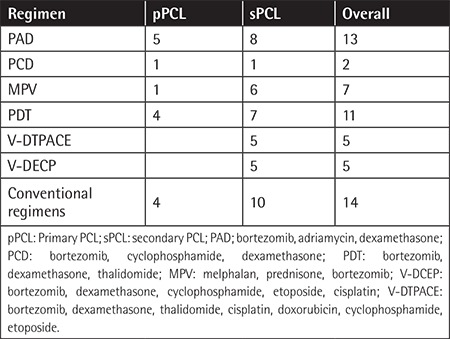
Therapeutic regimens.

**Table 3:**
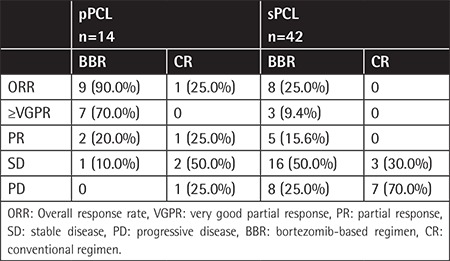
Response rate in patients treated with bortezomib-based regimens or conventional chemistry.

## References

[1] Tiedemann RE, Gonzalez-Paz N, Kyle RA, Santana-Davila R, Price-Troska T, Van Wier SA, Chng WJ, Ketterling RP, Gertz MA, Henderson K, Greipp PR, Dispenzieri A, Lacy MQ, Rajkumar SV, Bergsagel PL, Stewart AK, Fonseca R (2008). Genetic aberrations and survival in plasma cell leukemia. Leukemia.

[2] Kyle RA, Maldonado JE, Bayrd ED (1974). Plasma cell leukemia. Report on 17 cases. Arch Intern Med.

[3] Noel P, Kyle RA (1987). Plasma cell leukemia: an evaluation of response to therapy. Am J Med.

[4] Mina R, D’Agostino M, Cerrato C, Gay F, Palumbo A (2017). Plasma cell leukemia: update on biology and therapy. Leuk Lymphoma.

[5] D’Arena G, Valentini CG, Pietrantuono G, Guariglia R, Martorelli MC, Mansueto G, Villani O, Onofrillo D, Falcone A, Specchia G, Semenzato G, Di Renzo N, Mastrullo L, Venditti A, Ferrara F, Palumbo A, Pagano L, Musto P (2012). Frontline chemotherapy with bortezomib-containing combinations improves response rate and survival in primary plasma cell leukemia: a retrospective study from GIMEMA Multiple Myeloma Working Party. Ann Oncol.

[6] Katodritou E, Terpos E, Kelaidi C, Kotsopoulou M, Delimpasi S, Kyrtsonis MC, Symeonidis A, Giannakoulas N, Stefanoudaki A, Christoulas D, Chatziaggelidou C, Gastari V, Spyridis N, Verrou E, Konstantinidou P, Zervas K, Dimopoulos MA (2014). Treatment with bortezomib-based regimens improves overall response and predicts for survival in patients with primary or secondary plasma cell leukemia: analysis of the Greek Myeloma Study Group. Am J Hematol.

[7] Royer B, Minvielle S, Diouf M, Roussel M, Karlin L, Hulin C, Arnulf B, Macro M, Cailleres S, Brion A, Brechignac S, Belhadj K, Chretien ML, Wetterwald M, Chaleteix C, Tiab M, Leleu X, Frenzel L, Garderet L, Choquet S, Fuzibet JG, Dauriac C, Forneker LM, Benboubker L, Facon T, Moreau P, Avet-Loiseau H, Marolleau JP (2016). Bortezomib, doxorubicin, cyclophosphamide, dexamethasone induction followed by stem cell transplantation for primary plasma cell leukemia: a prospective phase II study of the Intergroupe Francophone du Myelome. J Clin Oncol.

[8] Musto P, Simeon V, Martorelli MC, Petrucci MT, Cascavilla N, Di Raimondo F, Caravita T, Morabito F, Offidani M, Olivieri A, Benevolo G, Mina R, Guariglia R, D’Arena G, Mansueto G, Filardi N, Nobile F, Levi A, Falcone A, Cavalli M, Pietrantuono G, Villani O, Bringhen S, Omede P, Lerose R, Agnelli L, Todoerti K, Neri A, Boccadoro M, Palumbo A (2014). Lenalidomide and low-dose dexamethasone for newly diagnosed primary plasma cell leukemia. Leukemia.

[9] Kyle RA, Durie BG, Rajkumar SV, Landgren O, Blade J, Merlini G, Kröger N, Einsele H, Vesole DH, Dimopoulos M, San Miguel J, Avet-Loiseau H, Hajek R, Chen WM, Anderson KC, Ludwig H, Sonneveld P, Pavlovsky S, Palumbo A, Richardson PG, Barlogie B, Greipp P, Vescio R, Turesson I, Westin J, Boccadoro M;, International Myeloma Working Group (2010). Monoclonal gammopathy of undetermined significance (MGUS) and smoldering (asymptomatic) multiple myeloma: IMWG consensus perspectives risk factors for progression and guidelines for monitoring and management. Leukemia.

[10] Fernández de Larrea C, Kyle RA, Durie BG, Ludwig H, Usmani S, Vesole DH, Hajek R, San Miguel JF, Sezer O, Sonneveld P, Kumar SK, Mahindra A, Comenzo R, Palumbo A, Mazumber A, Anderson KC, Richardson PG, Badros AZ, Caers J, Cavo M, LeLeu X, Dimopoulos MA, Chim CS, Schots R, Noeul A, Fantl D, Mellqvist UH, Landgren O, Chanan-Khan A, Moreau P, Fonseca R, Merlini G, Lahuerta JJ, Bladé J, Orlowski RZ, Shah JJ;, International Myeloma Working Group (2013). Plasma cell leukemia: consensus statement on diagnostic requirements, response criteria and treatment recommendations by the International Myeloma Working Group. Leukemia.

[11] Ramsingh G, Mehan P, Luo J, Vij R, Morgensztern D (2009). Primary plasma cell leukemia: a Surveillance, Epidemiology, and End Results database analysis between 1973 and 2004. Cancer.

[12] Sher T, Miller KC, Deeb G, Lee K, Chanan-Khan A (2010). Plasma cell leukaemia and other aggressive plasma cell malignancies. Br J Haematol.

[13] Colovic M, Jankovic G, Suvajdžic N, Milic N, Dorcevic V, Jankovic S (2008). Thirty patients with primary plasma cell leukemia: a single center experience. Medical Oncology.

[14] Pagano L, Valentini CG, De Stefano V, Venditti A, Visani G, Petrucci MT, Candoni A, Specchia G, Visco C, Pogliani EM, Ferrara F, Galieni P, Gozzetti A, Fianchi L, De Muro M, Leone G, Musto P, Pulsoni A;, GIMEMA-ALWP (Gruppo Italiano Malattie EMatologiche dell’Adulto, Acute Leukemia Working Party: coordinator Sergio Amadori) (2011). Primary plasma cell leukemia: a retrospective multicenter study of 73 patients. Ann Oncol.

[15] Mahindra A, Kalaycio ME, Vela-Ojeda J, Vesole DH, Zhang MJ, Li P, Berenson JR, Bird JM, Dispenzieri A, Gajewski JL, Gale RP, Holmberg L, Kumar S, Kyle RA, Lazarus HM, Lonial S, Mikhael J, Milone GA, Munker R, Nath R, Saccaro S, To LB, Vogl DT, Wirk B, Hari P (2012). Hematopoietic cell transplantation for primary plasma cell leukemia: results from the Center for International Blood and Marrow Transplant Research. Leukemia.

[16] Esparís‐Ogando A, Alegre A, Aguado B, Mateo G, Gutiérrez N, Bladé J, Schenkein D, Pandiella A, San Miguel JF (2005). Bortezomib is an efficient agent in plasma cell leukemias. Int J Cancer.

[17] van de Donk NW, Lokhorst HM, Anderson KC, Richardson PG (2012). How I treat plasma cell leukemia. Blood.

[18] Katodritou E, Verrou E, Gastari V, Hadjiaggelidou C, Terpos E, Zervas K (2008). Response of primary plasma cell leukemia to the combination of bortezomib and dexamethasone: do specific cytogenetic and immunophenotypic characteristics influence treatment outcome?. Leuk Res.

[19] Saccaro S, Fonseca R, Veillon DM, Cotelingam J, Nordberg ML, Bredeson C, Glass J, Munker R (2005). Primary plasma cell leukemia: report of 17 new cases treated with autologous or allogeneic stem-cell transplantation and review of the literature. Am J Hematol.

[20] Libby E, Candelaria-Quintana D, Moualla H, Abdul-Jaleel M, Rabinowitz I (2010). Durable complete remission of primary plasma cell leukemia with the bortezomib plus melphalan and prednisone (VMP) regimen. Am J Hematol.

[21] Lebovic D, Zhang L, Alsina M, Nishihori T, Shain KH, Sullivan D, Ochoa-Bayona JL, Kharfan-Dabaja MA, Baz R (2011). Clinical outcomes of patients with plasma cell leukemia in the era of novel therapies and hematopoietic stem cell transplantation strategies: a single-institution experience. Clin Lymphoma Myeloma Leuk.

[22] Ali R, Beksac M, Ozkalemkas F, Ozkocaman V, Ozkan A, Ozcelik T, Tunali A (2007). Efficacy of bortezomib in combination chemotherapy on secondary plasma cell leukemia. Leuk Lymphoma.

[23] Katodritou E, Terpos E, Delimpasi S, Kotsopoulou M, Michalis E, Vadikolia C, Kyrtsonis MC, Symeonidis A, Giannakoulas N, Vadikolia C, Michael M, Kalpadakis C, Gougopoulou T, Prokopiou C, Kaiafa G, Christoulas D, Gavriatopoulou M, Giannopoulou E, Labropoulou V, Verrou E, Kastritis E, Konstantinidou P, Anagnostopoulos A, Dimopoulos MA (2018). Real-world data on prognosis and outcome of primary plasma cell leukemia in the era of novel agents: a multicenter national study by the Greek Myeloma Study Group. Blood Cancer J.

[24] Mina R, Joseph NS, Kaufman JL, Gupta VA, Heffner LT, Hofmeister CC, Boise LH, Dhodapkar MV, Gleason C, Nooka AK, Lonial S (2019). Survival outcomes of patients with primary plasma cell leukemia (pPCL) treated with novel agents. Cancer.

[25] Jurczyszyn A, Castillo JJ, Avivi I, Czepiel J, Davila J, Vij R, Fiala MA, Gozzetti A, Grzasko N, Milunovic V, Hus I, Madry K, Waszczuk-Gajda A, Usnarska-Zubkiewicz L, Debski J, Atilla E, Beksac M, Mele G, Sawicki W, Jayabalan D, Charlinski G, Gyula Szabo A, Hajek R, Delforge M, Kopacz A, Fantl D, Waage A, Crusoe E, Hungria V, Richardson P, Laubach J, Guerrero-Garcia T, Liu J, Vesole DH (2019). Secondary plasma cell leukemia: a multicenter retrospective study of 101 patients. Leuk Lymphoma.

